# Ensemble model for estimating continental-scale patterns of human movement: a case study of Australia

**DOI:** 10.1038/s41598-021-84198-6

**Published:** 2021-02-26

**Authors:** Karen McCulloch, Nick Golding, Jodie McVernon, Sarah Goodwin, Martin Tomko

**Affiliations:** 1 Victorian Infectious Diseases Reference Laboratory, Royal Melbourne Hospital at The Peter Doherty Institute for Infection and Immunity, Parkville, VIC Australia; 2 Doherty Department, University of Melbourne, at The Peter Doherty Institute for Infection and Immunity, Parkville, VIC Australia; 3 School of Biosciences, The University of Melbourne, Parkville, VIC Australia; 4 Centre for Epidemiology and Statistics, Melbourne School of Population and Global Health, The University of Melbourne, Parkville, Australia; 5 Infection Modelling, Murdoch Children’s Research Institute, Parkville, Australia; 6 Faculty of Information Technology, Monash University, Caulfield, VIC Australia; 7 Melbourne School of Engineering, The University of Melbourne, Parkville, VIC Australia

**Keywords:** Statistics, Machine learning, Human behaviour

## Abstract

Understanding human movement patterns at local, national and international scales is critical in a range of fields, including transportation, logistics and epidemiology. Data on human movement is increasingly available, and when combined with statistical models, enables predictions of movement patterns across broad regions. Movement characteristics, however, strongly depend on the scale and type of movement captured for a given study. The models that have so far been proposed for human movement are best suited to specific spatial scales and types of movement. Selecting both the scale of data collection, and the appropriate model for the data remains a key challenge in predicting human movements. We used two different data sources on human movement in Australia, at different spatial scales, to train a range of statistical movement models and evaluate their ability to predict movement patterns for each data type and scale. Whilst the five commonly-used movement models we evaluated varied markedly between datasets in their predictive ability, we show that an ensemble modelling approach that combines the predictions of these models consistently outperformed all individual models against hold-out data.

## Introduction

Interaction models^1^ informed by population-scale mobility data are critical enablers for transport planning and logistics^2^, inequality and economical activity analysis^3^, urban planning and the provision of critical services, including health care and infectious disease epidemiology^4–7^, and other areas where the assessment of the impact of population-scale migratory flows is of importance. Human movement patterns are modelled, extracted and analyzed to better understand the intensities of interactions within a population^8^. A salient application of nuanced mobility pattern mining is infectious disease epidemiology, where the spread of infectious diseases may be facilitated by highly connected individuals or groups of individuals^9^. The identification of (sub)populations at risk may enable better, more targeted public health response, or aid understanding of the contributions of such individuals to epidemic spread^10–12^. Recent work has focused on estimating the impact of restricting mobility as an intervention to slow the spread of SARS-CoV-2^13^. Research linking human movement patterns with the socio-economic status of sub-populations, however, also shows that these relationships are complex and may vary between cities or regions^14^. Yet, too often do population-scale models rely on simplistic mathematical assumptions and lack the understanding of the data that goes into the movement models to predict movement flows.

In practice, the problem of model choice is further confounded with the choice (or availability) of the data used to fit the model. Call Detail Records (CDR) from mobile phones^6,15–18^, population census data^11,15,19^ and global positioning satellite (GPS)^16,20–22^ data are currently the most widely available data applicable to studies of human movement (historically, other data have been explored, including bank notes^23^). The census is the most comprehensive and costly source of mobility data that strives to capture the population of a country, but has to sacrifice temporal resolution and nuance (data are collected on a single census day typically every five years). Mobility can be inferred from a single census question related to a person’s journey to work which requires information on their location of usual residence and workplace on the day the census was conducted. CDR-based data are available only for a sub-population of phone users, typically those subscribed to one (out of multiple) mobile providers. The locations of placed or received calls are captured based on the nearest cell tower, at a relatively coarse and variable spatial resolution which is entirely dependent on the distribution of cell towers. Trips can then be inferred, but their purpose remains unknown. With the shift of mobile users to voice over IP and chat communication, these data are increasingly less representative of movement patterns. However, the use of GPS data collected from satellite navigation applications to infer human movement is becoming increasingly popular. Detailed GPS data tracking via mobile applications capture only small sub-populations of users, but come at a much finer spatial granularity and for all types of trips^20,24^. Yet, how well do distinct data sources capture population-scale mobility? The choice of dataset determines the success of the statistical model derived from it, yet all too often the choice is only driven by data availability. Determining what type of data is most appropriate to capture the mobility behaviour across a specific geographical region is treated as a secondary issue^11,21^.

Population mobility models can generally be divided into two groups, those that consider movement frequency (i.e. the number of trips) between two locations to be a decreasing function of distance (gravity models^15,25^) and those that assume the number of potential destinations between two locations determines movement frequency (intervening opportunities model^26^)^21^. However, more recently developed radiation models^15,27^ fall in between these two groups of models and utilise a 2-step process to estimate movement frequency between two locations. In step 1, all locations are considered as possible destinations and are ranked depending on opportunities (which are proportional to the location population). In step 2, the location closest to the origin with the highest rank is chosen as the preferred destination^15^. Gravity models have been used to estimate large scale migration patterns across countries^11^. The radiation model has been shown to outperform the gravity model on occasion, including predicting movement patterns within a state and across country scale^15^. However, both model types have been shown to inadequately describe population movement in some settings, i.e. low income countries^12^.

A form of ensemble model was first introduced by Wolpert^28^ as stacked generalisations which are motivated by the goal of reducing the generalisation error (or the out-of-sample error). The idea was later formalised by Breiman^29^ and the theoretical background was developed by Van der Laan et al.^30^ Stacked generalisation involves training a new model to combine predictions of existing models in order to improve predictive performance^31^. During this process the new model learns weights to assign to each existing model based on expected performance. This improves on model averaging processes in which each existing model would be weighted equally. The use of ensemble model approaches aims to shift thinking from selecting the single best available model to deriving an improved model which incorporates information from all available models.

This paper considers the combined challenges of data scales and model types to infer mobility patterns across Australia. Our work is motivated by the need to understand population mobility as a necessary step in the assessment of access to health care services in Australia which varies widely across the continent. We address the need for movement models capturing mobility flows across large geographic areas but representative across spatial scales. The models should capture short distance and long distance travel, adapt to highly heterogeneous space use and diverse geographies. As currently no single model performs consistently best across such varied conditions, we hypothesize that a weighted, locally adaptive combination of each of these models in an ensemble model will better capture the observed movement patterns. Models are trained on data obtained from the Australian Bureau of Statistics (ABS) Census (a snapshot view of the population mobility through their journeys to work between statistical areas)^32^ and a dataset collected by the provider of a smartphone-based GPS satellite navigation application. This dataset captures a smaller sample of the population, with a richer set of journeys capturing diverse, yet unspecified, travel needs. We compare model predictions across all models, both datasets, and both spatial scales. In doing so we highlight the strengths and weaknesses in predictive capacity of currently available movement models. Finally, we implement an ensemble model that leads to improved, more accurate predictions of movement flows across different geographical scales.

## Results

**Figure 1:**
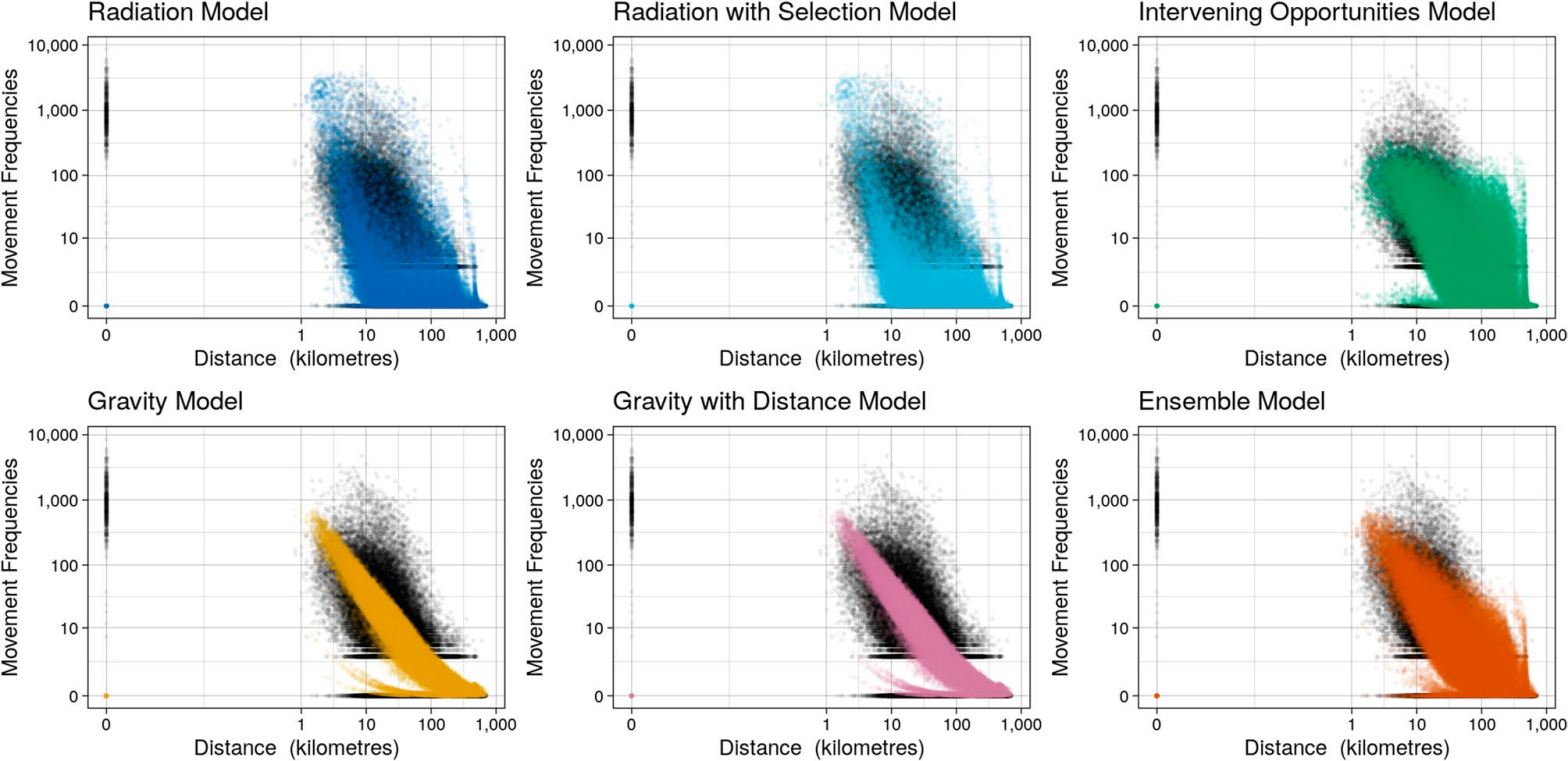
Comparison of predicted frequency of movements by distance (kilometres) between pairs of locations for each model type, based on journey to work Census data. Black points show the raw movement frequencies that were used to train each model. Coloured points show the resulting model predictions. Movement frequency and distance are shown on a log-scale to better highlight differences between model predictions. This figure was produced using the R^33^ package ggplot2^34^.

**Figure 2:**
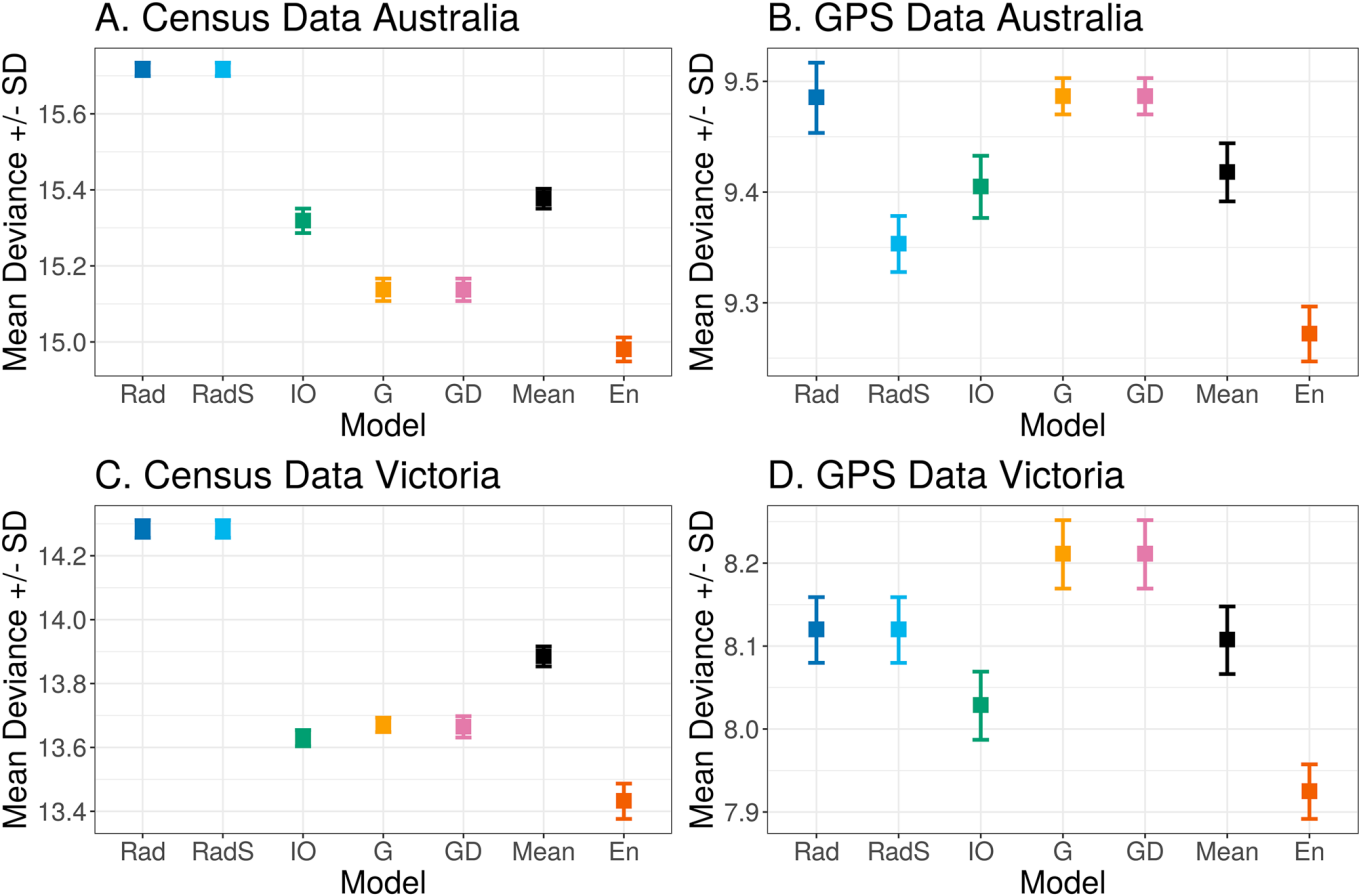
Comparison of mean deviance between models for each dataset and geographic scale. Legend: G = gravity model; GD = gravity with distance model; Rad = radiation model; RadS = radiation with selection model; IO = intervening opportunities model; En = ensemble model; Mean = mean deviance across all model types. Note the y-axis is on a log scale and the absolute value of the deviance is not comparable between datasets. The deviance is a measure of how well a given model predicts the test data (which was not used to train the models) relative to the other models for a given dataset. The error bars show how the deviance varied for each model type across the 5-folds. This figure was produced using the R^33^ package ggplot2^34^.

The ABS journey to work census data contains counts of individuals travelling between their region of home residence and regions of place of work on the census day, where regions were defined at Statistical Area 2 (SA2) granularity. In Fig. 1 we illustrate the differences in predictive capacity of the five models described in the literature (herein referred to collectively as the base models), using journey to work census data for the Australian state of Victoria. From Fig. 1, it appears that the intervening opportunities model captures more of the observed movement patterns at the state scale than the other base models. The radiation models appear to capture the breadth of the observed data but the intensity of movement predictions is misplaced, i.e., radiation models over-predict the lower frequencies of movement over long distances. In contrast, the gravity models predictions cover a narrow band of the observed data but with higher intensity of predictions. By assigning weights to the predictions generated by each of the base models, the ensemble model is able to capture a wider range of these features. The differences in predictive performance for each of these models is further reflected in Fig. 2C where it is clear that the ensemble model outperforms all base models. The Poisson deviance reported in Fig. 2 is derived by comparing model predictions made on out-of-sample data to the corresponding observed data and can be interpreted as an estimate of model performance relative to other models trained on the same data. A lower deviance indicates a better model fit. Similar patterns were seen for predictions generated from the whole of Australian census data (see Figure S1, Supplementary Material). To aid comparison of base model predictions we provide an alternative visualisation of Fig. 1 in Figure S2 of the Supplementary Material.

Anonymised GPS data from a smartphone based satellite navigation application were aggregated to provide population level travel flow frequencies between SA2 regions. The observed movement patterns for the GPS data differ the census data (Figures S3 to S6 of Supplementary Material). This is not surprising given the inherently different nature of the data sources, for example the GPS data sample size was much smaller and the maximum observed movement frequency is about half of that seen in the census data. There is more variation in the Poisson deviance estimates for the GPS data (Fig. 2B and D) than the census data. These data encompass a variety of movement flows as the reasons for individual trips is unknown, this is a likely source of some of the variation seen. Therefore, the trained models struggle to capture a wider variety of movement patterns to reflect the input data. Interestingly, out of the five base models the intervening opportunities model performs best at the smaller geographic state scale (Victoria) across both the ABS census journey to work data and the GPS data. At the continental scale, out of the five base models, the gravity models perform best for the ABS census journey to work data, whereas the radiation with selection model performs best for the GPS data. There are other similarities across geographic scales for the datasets, in general the gravity and intervening opportunities models outperform the radiation models for the census data across scales, while the radiation models and intervening opportunities models generally perform better for the GPS data. These results suggest that each dataset is capturing different movement patterns (i.e. purpose of trip) and support the idea that it is necessary to consider multiple types of movement data in order to best capture population movements. These results also highlight that when it comes to population movements there is no single model that optimally fits different data sources. By combining information from each of the base models into an ensemble model we are able to capture more of the observed movement patterns which would have otherwise been missed. The derived ensemble model consistently out-performs the widely used and reported models in the literature for each dataset and both geographic scales (state of Victoria and continental Australia). This can be seen clearly in Figs. 1 and 2 (and Figures S1 to S4 of Supplementary Material). The parameters for the ensemble models trained on Victorian and Australian census and GPS data are provided in the Supplementary Material. In addition to this we provide the Poisson deviance results for each model and dataset from the 5-fold cross validation (see Methods Section for further details) process.

**Figure 3:**
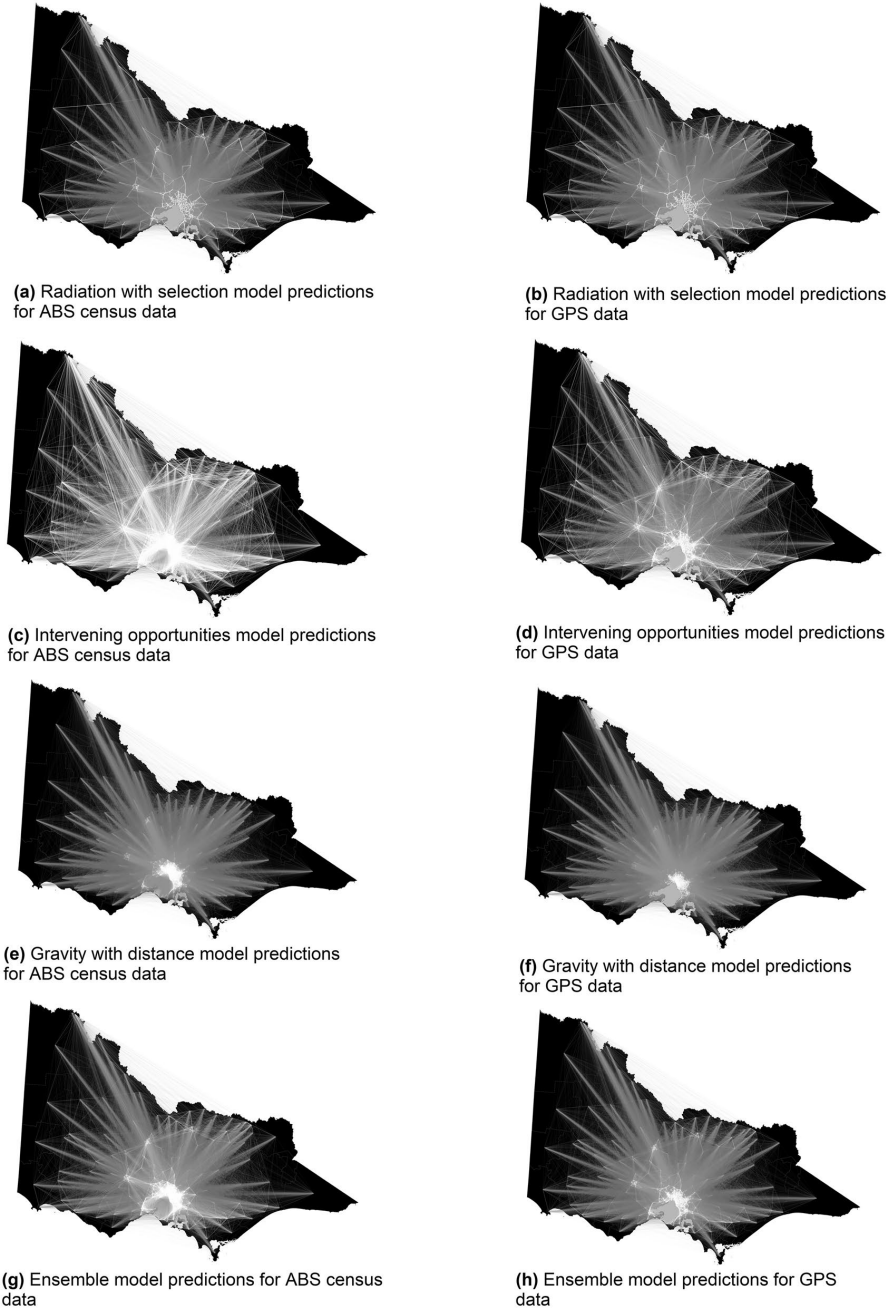
Range and intensity of interaction in Victoria revealed through different model types using the ABS census journey to work data (left column) and GPS data (right column). Movement predictions from each model were scaled to be between 0 and 1 to aid visual comparison. This range is mapped to line thickness, opacity and brightness (from black to white), where vivid thick white lines represent high intensity movement and low intensity movement is shown via faint thin darker lines. The Gravity and radiation model prediction maps are omitted as they are visually similar to the gravity with distance and radiation with selection model predictions respectively. The maps in this figure were produced using the R^33^ package ggplot2^34^ (see Visualisation of predictions section in Methods for further detail). Preview^35^ and Paint 3D^36^ were used to combine the maps into one image and add the text.

**Figure 4:**
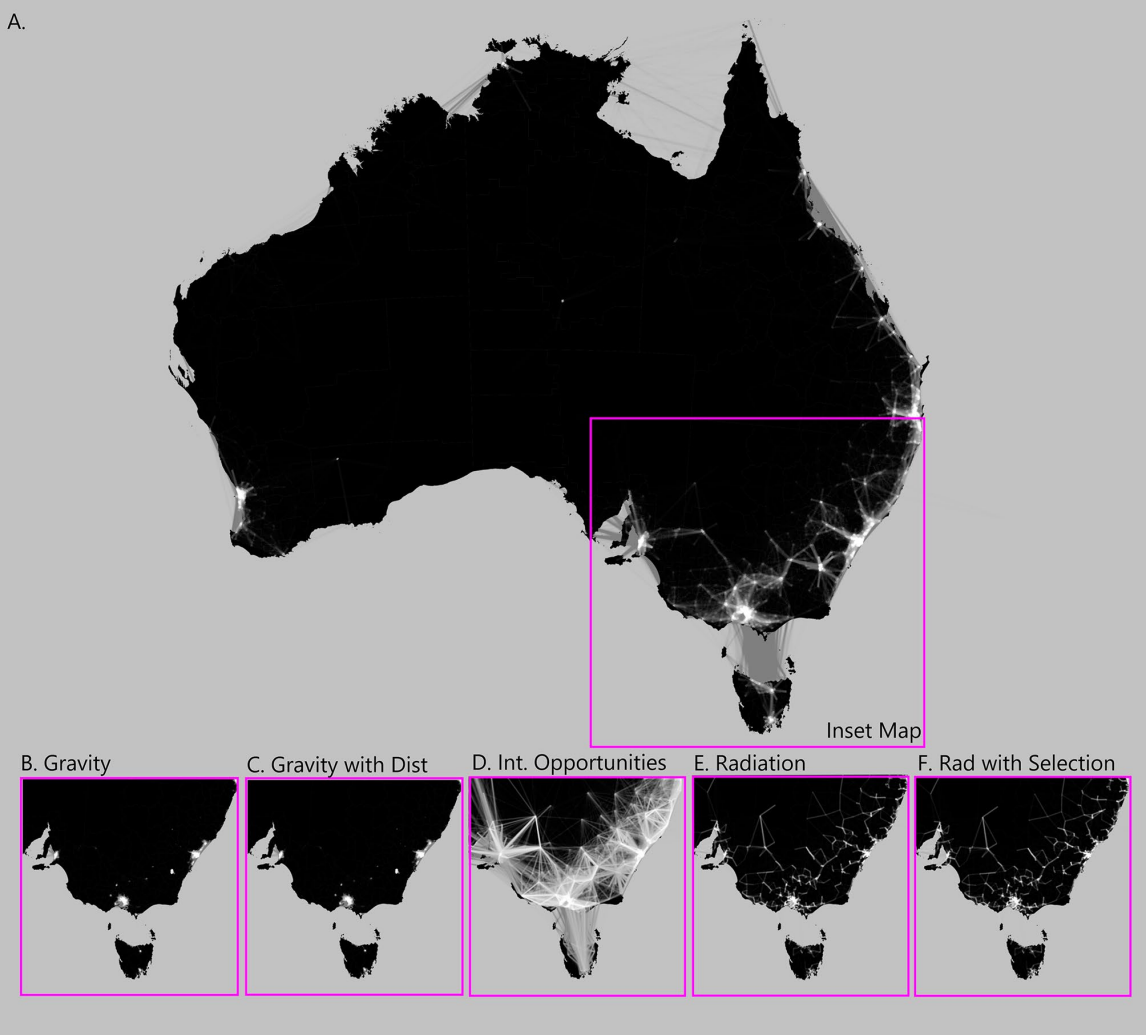
Ensemble model predictions for Australia using ABS census data (**A**). Inset maps for gravity (**B**), gravity with distance (**C**), intervening opportunities (**D**), radiation (**E**) and radiation with selection (**F**) models respectively all trained using ABS data. Movement predictions from each model were scaled to be between 0 and 1 to aid visual comparison. Vivid thick white lines represent high intensity movement, whilst faint thin darker lines represent low intensity movement. The maps in this figure were produced using the R^33^ package ggplot2^34^ (see Visualisation of predictions section in Methods for further detail). Preview^35^ and Paint 3D^36^ were used to combine the maps into one image, overlay the squares and add the text.

Differences in movement patterns and intensities predicted by the base models at the state scale for each dataset are illustrated in Fig. 3. These graphs further highlight the importance of selecting the best fitting model, as the patterns of predicted movement vary significantly between models trained on the same dataset. We can also compare patterns across the two datasets for the same model type. Generally, across the model types the intensities and patterns that have been predicted for the census data are more defined than those for the GPS data. The Melbourne Metropolitan region is clearly visible in all results. The most notable difference in predictions is by the intervening opportunities model on the census (Fig. 3c) and GPS (Fig. 3d) datasets. The census data predicts higher intensities of movement across long distances, while the GPS data better distinguishes between short to medium distance movement patterns. This is also reflected in Fig. 1 and Figure S3. The movement pattern in Fig. 3g and h illustrate the ensemble model predictions for ABS census data and GPS data respectively. The resulting weights of the ensemble model for the state scale census data indicate that the radiation and radiation with selection model predictions are not very informative. The radiation models predictions are very similar for the census data and they are weighted in such a way that the information provided will almost cancel out (Table 1) when making predictions utilising the ensemble model. Therefore, the ensemble model for census data is influenced more by the intervening opportunities and gravity models. Similarly, the ensemble model for the state scale GPS data is influenced more by the intervening opportunities and radiation model predictions. These differences further reflect the different types of movement patterns being captured within each dataset. Figure 4 illustrates the differences in predictive capacity of the base models compared to the ensemble model trained on the ABS census data at the continental scale. The gravity models (Fig. 4B and C) highlight major population hubs such as capital cities across the eastern jurisdictions in Australia with minimal movement flows outside of these hubs. In contrast to the gravity model predictions, the intervening opportunities model (Fig. 4D) predicts long range movement flows at high intensities. The ensemble model predictions clearly highlight the population hubs of the capital cities in addition to smaller regional hubs. Figures 3 and 4 highlight the importance of selecting the most appropriate dataset and geographic scale to predict movement patterns and intensities relevant to the questions being asked.

## Discussion

We have illustrated that an ensemble model with learned weights from currently available movement models outperforms any individual base model in terms of predictive capacity. We hypothesized that different interaction models are suitable for modelling different parts of the specified geographic region. This cannot be simply achieved by averaging across the models (Fig. 2, *Mean* results) but it can be achieved by utilising an ensemble model. The ensemble model learns from the predictions made by the base models and assigns weights to each set of predictions to reflect which models were the most accurate. The resulting ensemble model predictions better reflect the underlying patterns of movement displayed by the data on which the base models were trained. Furthermore, interactions across large, heterogeneous regions are captured better by the ensemble model. In this study we used Australia as our case study due to the diverse landscape, however this methodology can easily be applied to any other region and landscape.

We have shown that no single model can comprehensively capture the interactions across large, heterogeneous regions. This highlights the importance of considering different types of interactions (predicted by currently available movement models) through the use of ensemble models. In addition, we have also shown that the same type of model trained on different data will lead to different models and as such it is important to consider the type of data required for estimating movement flows in different contexts.

For some applications and geographical scales of inquiry, the accuracy of the flows predicted by the gravity and intervening opportunities models may be sufficient (e.g., freight transportation, job seeking behaviour). Recent research has, however, focused on identifying gaps in the formulation and application of quantitative models of movement to improve the accuracy and predictive power beyond levels achieved thus far. The radiation model was proposed by Simini et al.^15^ as an alternative to the gravity model which may under certain conditions lead to more accurate predictions and has been shown to outperform the gravity model in predicting movement flows across multiple temporal scales. These mathematically elegant and computationally accessible movement models have enabled a widespread proliferation of mobility studies providing a coarse understanding of the population dynamics from national to metropolitan scales. Yet, we argue that the majority of these studies applied the models without attempting to understand their nuanced assumptions, their applicability across geographies and scales, and the errors in the predictions. Population-level movements differ by the motivation and need travel satisfies (e.g., journey to work or for leisure, or flight from a natural or human hazard), by mode of transport, the geographies covered (cities, coastal plains or mountainous areas), and the temporal dependencies of consecutive travel, each presenting complications for these fundamentally elegant, yet simplistic statistical models.

More recent research in this field^4,12,27^ has emphasized the need to validate competing models and proposed a variety of model extensions, each with uncertain transfer-ability to different study areas with distinct space-use patterns or to other applications (transport logistics, population migration and commuting populations). A rigorous comparison of movement flows predicted using different models by Lenormand et al.^19^ highlights that the same input data must be used across models for meaningful comparison. In their study, the gravity models with an exponential decay distance function outperformed the intervening opportunities model, highlighting how a person’s home location presents a more important parameter than the number of intervening opportunities (work locations). Yet, the ability to tailor and fit nuanced, sophisticated, and locally adapted models^37^ is usually either not possible (due to lack of data, and the urgency to model a dynamically evolving phenomenon) or not easily transferable to a different geography or context. A possible departure is the approach of Kraemer et al.^7^, researching generic parameterisations of models of human mobility. More recent research has focused on developing models to improve the predictability of large-scale mobility by utilising a data-driven preferential attachment model^18^.

Ensemble models are valuable tools that can be used to improve predictive accuracy based on existing models, however a key limitation is the required computational power to train base models and carry out cross validation. Given the improvements in computational power since these methods were developed, they are likely to become increasingly popular in future. The Poisson deviance is a measure of how well the model predicts to the out-of-sample data, for this reason it cannot be used as a metric to compare across data sets and can only be used to compare relative model fits on the same data. The ABS census and GPS data both represent different inherently biased samples of population movements. A clear limitation of the census data is that it excludes population movements for those who are not currently employed, in addition the census is a single day sampling event and thus it is only reflective of the population that was employed on the day the census was conducted. The GPS data was sourced from a navigation application and as a result would capture different types of movement, restricted to those who used the navigation application. Therefore, the ABS census data may be reflective of the general working population movements whereas the GPS data will likely only reflect a portion of the population movements. Future work should consider expanding the GPS data set to see if patterns observed in this study hold true for a larger sample.

Our findings can be used to inform epidemiological research across country scale. This research was largely motivated by the need to understand how populations travel in order to access relevant health care. As such, firstly deriving models from appropriate data that can be utilised to predict movement flows between regions more accurately is of key importance. Secondly, combined with appropriate data these movement models can be further utilised to determine a measure of accessibility to relevant health care. Measuring accessibility to health care services also requires consideration of different sub-populations affected, which will again require appropriate use of data.

Standard movement model frameworks (such as the well known gravity and radiation models) take into account the Euclidean distance between the origin region and the destination region, while this can provide some level of insight into overall movement patterns it is not a realistic assumption for longer range movement within Australia. Due to the diverse landscape of Australia, it is necessary to consider the road distance or the road travel time between two regions and not simply the Euclidean distance which does not take into account the diversion of roads around mountain ranges, national parks or deserts. An extension to this work would be to take into account different measures of time and distance, as well as to look at changing patterns of movement over time to see if the same results hold as presented here. Furthermore, finding ways to integrate large spatial scale movement data (i.e. census) with more granular movement data (i.e. GPS) to derive a single model utilising both data types is another crucial way the analyses presented here could be extended upon.

## Methods

### Data sources

#### Census data

The Australian Bureau of Statistics (ABS) Census data are collected every 5 years and are publicly available online. Within the Employment, Income and Unpaid work section, the journey to work dataset^32^ contains counts of individuals travelling between their region of home residence and regions of place of work on the census day. We use these data for regions at Statistical Area 2 (SA2) granularity a) within Victoria and b) across the whole of Australia, based on findings of the 2011 census. The ABS define SA2 regions to have a population size between 3,000 and 25,000, with the mean population size across all SA2 regions equal to 10,000. This results in varied spatial scales of SA2 regions across Australia, with urban regions being more populated and smaller than those less populated regions in remote areas.

#### Global positioning satellite data

Anonymised GPS data from approximately 150,000 unique Australian users of a smartphone-based satellite navigation application capturing individual trips were processed and aggregated to provide population level travel flow frequencies between Statistical Area 2 (SA2) regions from January to October 2016 for a) Victoria and b) the whole of Australia. Aggregated trip frequencies over the entire dataset coverage period were processed to obtain an origin-destination matrix containing the average daily movement between SA2 regions. Each matrix was then normalised to produce a matrix of intensity of contact between origins and destinations. The raw data are not publicly available, however the processed origin - destination matrices are available upon request.

A visual comparison of the observed ABS census and GPS data can be found in Figures S5 and S6 of the Supplementary Material.

### Movement models

Here, we describe the parameterised movement models used to fit the two described data sources and to predict patterns of population movement across Australia.

1. Gravity Model: The gravity model assumes that the number of individuals moving between locations (origins and destinations) is proportional to a product of the population size of each location, and is inversely proportional to distance. The gravity model assumes that the magnitude of the number of trips,$T_{ij}$, between locations *i* and *j* is defined by:
  $$\begin{aligned} T_{ij} \propto \dfrac{m_i n_j}{r_{ij}} \end{aligned}$$
  where $m_i$ is the population size of the origin, $n_j$ is the population size at the destination and $r_{ij}$ is the distance between *i* and *j*. We use a common functional form of the gravity model which is defined as follows:
  $$\begin{aligned} T_{ij} = \theta \dfrac{m_i^\alpha n_j^\beta }{r_{ij}^\gamma } \end{aligned}$$
  where $T_{ij}$ is the number of individuals moving between location *i* and *j*, $m_i$ represents the population at the origin location, $n_j$ is the population at the destination location and $r_{ij}$ is the distance between the two locations, $\theta$ is a constant.
  The parameters $\alpha$ and $\beta$ tune the dependence of dispersal on the origin and destination populations respectively^15,38^.
2. Gravity with Distance cut off: This model is a modification of the gravity model which allows short and long trips to be fitted separately, resulting in more accurate predictions^10^. The probability of travelling between two locations within a given distance, $\delta$, is different to the probability of travelling between two locations which are separated by a distance greater than $\delta$. Therefore, if $r_{ij} < \delta$ we have
  $$\begin{aligned} T_{ij} = \theta _1 \dfrac{ m_i^{\alpha _1} n_j^{\beta _1}}{r_{ij}^{\gamma _1}} \end{aligned}$$
  and if $r_{ij} > \delta$ we have
  $$\begin{aligned} T_{ij} = \theta _2 \dfrac{ m_i^{\alpha _2} n_j^{\beta _2}}{r_{ij}^{\gamma _2}} \end{aligned}$$
  where $\theta _1$ and $\theta _2$ are proportionality constants. The exponents $\alpha$ and $\beta$ tune the dependence of dispersal on the origin and destination population sizes ($m_i$ and $n_j$ respectively) and the distance between two regions, $r_{ij}$^38^.
3. Original Radiation: The (original) radiation model generally assumes the rational of job selection. It follows the general rule that the number of employment opportunities in each region is proportional to its resident population, assuming full employment (that is the number of people in a region is equal to the number of jobs in that region). Moreover, the individuals in each region choose the closest job to their home. The average movement flow from location *i* to location *j* predicted by the radiation model is defined by:
  $$\begin{aligned} T_{ij} = \theta \dfrac{m_i n_j}{(m_i + s_{ij}) (m_i + n_j + s_{ij})} \end{aligned}$$
  where $m_i$ is the population at the origin and $n_j$ at the destination, $s_{ij}$ denotes the total population in a specified radius around population centres $m_i$ and $n_j$, and $\theta$ is the proportion of the population in region *i* that commute^15,38^. In this study, the radiation model parameters are estimated based on information about opportunities in each region.
4. Radiation with Selection: This model is a combination of the original radiation model (above) and intervening opportunities model (see below). The average movement from region *i* to region *j* according to the radiation model with selection is defined by:
  $$\begin{aligned} T_{ij} = \theta \dfrac{(1 - \frac{\lambda ^P}{P} )( 1 - \frac{\lambda ^Q}{Q} )}{ ( 1 - \frac{\lambda ^R}{R} )} \end{aligned}$$
  where $\theta$ is the proportion of population *i* that commute, *P* is the population at the origin location *i* and *Q* at the destination location *j*, *R* denotes the total population in a radius $\lambda$ around population centres $P_i$ and $Q_j$^5,15,27,38^.
5. Intervening Opportunities: Stouffer^26^ formulated the intervening opportunities model based on the idea that distance and mobility are not directly related as suggested by the gravity model. The reasoning behind this is formed by the idea that the decision to make a trip is related to the relative accessibility of opportunities that satisfy the objective of the trip^21^. That is, the higher the number of intervening opportunities between two points, the lower the number of people travelling the whole distance between those two points. This results in the following definition:
  $$\begin{aligned} T_{ij} \propto \dfrac{n_j}{(s_{ij} + m_i) } \end{aligned}$$
  where $n_j$ is the total population in location *j* and ($s_{ij}$ + $m_i$) is the total population in locations between *i* and *j*^26^. This formulation of the model was redefined as a stochastic approach by Schneider in 1959 as explained in Simini et al.^15^. The probability that an individual in location *i* travels and ends their trip in location *j* is equal to the probability that location *j* contains an acceptable destination and that an acceptable destination closer to the origin location *i* has not been found. Therefore, the number of trips between location *i* and *j* is defined by:
  $$\begin{aligned} T_{ij} \propto e^{-\lambda (s_{ij} + n_i)^\theta } - e^{-\lambda (s_{ij} + n_i + n_j)^\theta } \end{aligned}$$
  where $e^{-\lambda }$ is the probability that a single opportunity is not sufficiently attractive as a destination^15^. We make use of this intervening opportunities model defined by^15^ for the purposes of this study.

As the above movement models do not allow for movements within the same region (i.e. self loops), all predicted values for which the origin and destination location are the same were set to zero.

#### Ensemble movement model

An ensemble movement model is derived based on the hypothesis that combining information from each type of movement model outlined above will produce more robust predictions of movement flows. We derive a type of ensemble model referred to as a stacked model, an idea first introduced by Wolpert^28^ as stacked generalizations. Breiman^29^ later introduced the concept of stacked regressions, which developed on Wolpert’s ideas and formalized a way to generate improved prediction accuracy by forming linear combinations of different predictors which were trained on the same dataset. Below we describe our ensemble model which utilises a Poisson generalized linear model (GLM)^39^ as the ‘stacker’ to derive more robust predictions of movement flows between locations using the predictions generated from each of the above models as our input data.

Let $\Lambda _a(i,j)$ be the predicted movement flow matrix derived from model *a*, where $(i,j) \in \{1 \ldots z\}$ and *z* is the number of locations of interest. Then $\lambda _{ak}$ is the corresponding vectorized form of $\Lambda _a$ with $k=1 \ldots z^2$. In the following $a=1$ denotes predictions generated from the gravity model; $a=2$ denotes gravity with distance model; $a=3$ denotes radiation model; $a=4$ denotes radiation with selection model and $a=5$ denotes intervening opportunities model. Let $y_k$ be the ensemble predicted movement flow and $\mu _k$ be the expected value of $y_k$. We estimate $\mu _k$ by deriving a Poisson GLM which takes the following form

$$\begin{aligned} log(\mu _k)= & {} \beta _0 + log(\lambda _{1k}^{\beta _1}) + log(\lambda _{2k}^{\beta _2}) + log(\lambda _{3k}^{\beta _3}) + log(\lambda _{4k}^{\beta _4}) + log(\lambda _{5k}^{\beta _5}) \\= & {} \beta _0 + \beta _1 log(\lambda _{1k}) + \beta _2 log(\lambda _{2k}) + \beta _3 log(\lambda _{3k}) + \beta _4 log(\lambda _{4k}) + \beta _5 log(\lambda _{5k}) \end{aligned}$$

where each of the $\beta _i$ coefficients (also referred to as weights) are fitted to the input data. Then, $E[y_k] = \mu _k$. The estimated weights for the ensemble models for each dataset are provided in Table 1. After transforming the vector $\mu _k$ back into a matrix of size $z \times z$, we obtain the matrix for the predicted movement flows between locations *i* and *j*

$$\begin{aligned} E[y(i,j)]= \mu (i,j). \end{aligned}$$

**Table 1:** Ensemble model weights.

Parameter	Ensemble model			
	Census Victoria data	Census Australia data	GPS Victoria data	GPS Australia data
$\beta _0$	0.9096	0.9442	$-$0.0021	0.3163
$\beta _1$	0.3215	0.5347	2897.8930	1.1379
$\beta _2$	$-$0.0036	$-$0.0687	$-$2897.9680	$-$0.8193
$\beta _3$	821.6171	25.7575	$-$93.8892	$-$0.0894
$\beta _4$	$-$821.4218	$-$25.5504	94.1929	0.6769
$\beta _5$	0.3701	0.1945	0.3829	0.1419

Parameter description: $\beta _0$ model intercept, $\beta _1$ gravity model weight, $\beta _2$ gravity with distance model weight, $\beta _3$ radiation model weight, $\beta _4$ radiation with selection model weight and $\beta _5$ intervening opportunities model weight.

### Model parameterisation and prediction

We parameterise the five movement models described above by fitting each to the two observed population movement flow datasets (ABS and GPS) separately. Model fitting was performed using the movement package^38^ in R^40^. The fitting process results in estimates for each of the tunable parameters specified in models (1) to (5) above. Parameters for each model are optimised based on log likelihoods against the observed data assuming a poisson sampling distribution, as in the ensemble model. The Broyden-Fletcher-Goldfarb-Shanno (BFGS)^41^ method is utilised to carry out the parameter optimisation process.

#### Cross validation

A *k*-fold cross validation with $k=5$ folds has been used to assess how well the above models predict movement. First, each dataset (ABS and GPS) is divided up into 5 folds (or sections). We then parameterised the movement models (described by (1) to (5) above) by training them using 4 folds (or $80\%$ of the data). Then the parameterised models were used to predict the witheld fold (or remaining $20\%$ of the data) in order to evaluate prediction accuracies of the models. This process was repeated until each of the 5 folds had been used as the witheld data. The 5-fold cross validation was also carried out for the ensemble model. This approach was implemented using the Caret^42^ R package.

After training the model, the accuracy of predictions was evaluated by computing the Poisson deviance. Poisson deviance allows measurement of the error between the observed and predicted values and thus enables assessment of how well each individual model predicts observed movement intensities between locations. It is important to note that the Poisson deviance provides a measure of relative fit. This means that values of the Poisson deviance can be compared across each fold for the same model but values cannot be compared between models. Tables S1 to S4 in the Supplementary Material give the Poisson deviance for the comparison of predicted and observed movement flows for each fold in the cross validation for each combination of model type and data source. Tables S5 to S8 in the Supplementary Material provide parameter estimates generated from 5-fold cross validation for each of the model types.

Once each model was parameterised, we used it to predict the movement flows and evaluate the accuracy of the predictions against the observed data. Even though we fitted the models using frequency travel data between SA2 regions in Australia (which can be large geographical areas), the final parameterised models can be utilised to predict the likelihood of movement flows between smaller geographical areas of any size, with the caveat that the location and population size of the origin and destination areas is still required.

### Visualisation of predictions

To aid geographical visualisation, the predicted movements for each model were put into an origin - destination matrices and scaled to ensure all predictions were between 0 and 1. This allowed better comparison of different model predictions within and between datasets. Figures 1 to 4 were produced using the R^33^ package ggplot2^34^. Preview^35^ and Paint 3D^36^ were used to combine the maps into one image, overlay the squares and add the text on Figs. 3 and 4. Data used in Figs. 3 and 4 consist of predictions generated from the individual models and the ensemble model, which utilised ABS census and GPS data (see Methods section for details) as inputs into the models. The geographic boundaries of Australia and the state of Victoria for the maps in Figs. 3 and 4 were sourced from the ABS and mapped using the Geocentric Datum of Australia 1994 (GDA94) coordinate reference system.

## Supplementary information

Supplementary files

## Data Availability

The Australian Bureau of Statistics census journey to work data is freely available to download from their website. The raw GPS data utilised are not publicly available, however the processed origin - destination matrices are available upon request. All model predicted origin-destination matrices are available upon request.
