# Supplementary material for: Ensemble model for estimating continental-scale patterns of human movement: a case study of Australia
Source: Sci Rep. 2021 Feb 26;11:4806. doi: 10.1038/s41598-021-84198-6 (PMC7910534; doi:10.1038/s41598-021-84198-6)
Supplement: Supplementary file 1 — Supplementary files [file 41598_2021_84198_MOESM1_ESM.pdf]

SUPPLEMENTARY MATERIAL
Ensemble model for estimating continental-scale patterns of human movement: a case study of Australia

Karen McCulloch[1,2,*], Nick Golding[3], Jodie McVernon[1,4,5], Sarah Goodwin[6], and Martin Tomko[7,*]

[1]Victorian Infectious Diseases Reference Laboratory, Royal Melbourne Hospital, at The Peter Doherty Institute for Infection and Immunity, Parkville, VIC, Australia.
[2]Doherty Department, University of Melbourne, at The Peter Doherty Institute for Infection and Immunity, Parkville, VIC, Australia.
[3]School of Biosciences, The University of Melbourne, Parkville, VIC, Australia.
[4]Centre for Epidemiology and Statistics, Melbourne School of Population and Global Health, The University of Melbourne
[5]Infection Modelling, Murdoch Children's Research Institute
[6]Faculty of Information Technology, Monash University, Caulfield, VIC, Australia.
[7]Melbourne School of Engineering, The University of Melbourne, Parkville, VIC, Australia.
[*]Corresponding authors: karen.mcculloch@unimelb.edu.au, tomkom@unimelb.edu.au

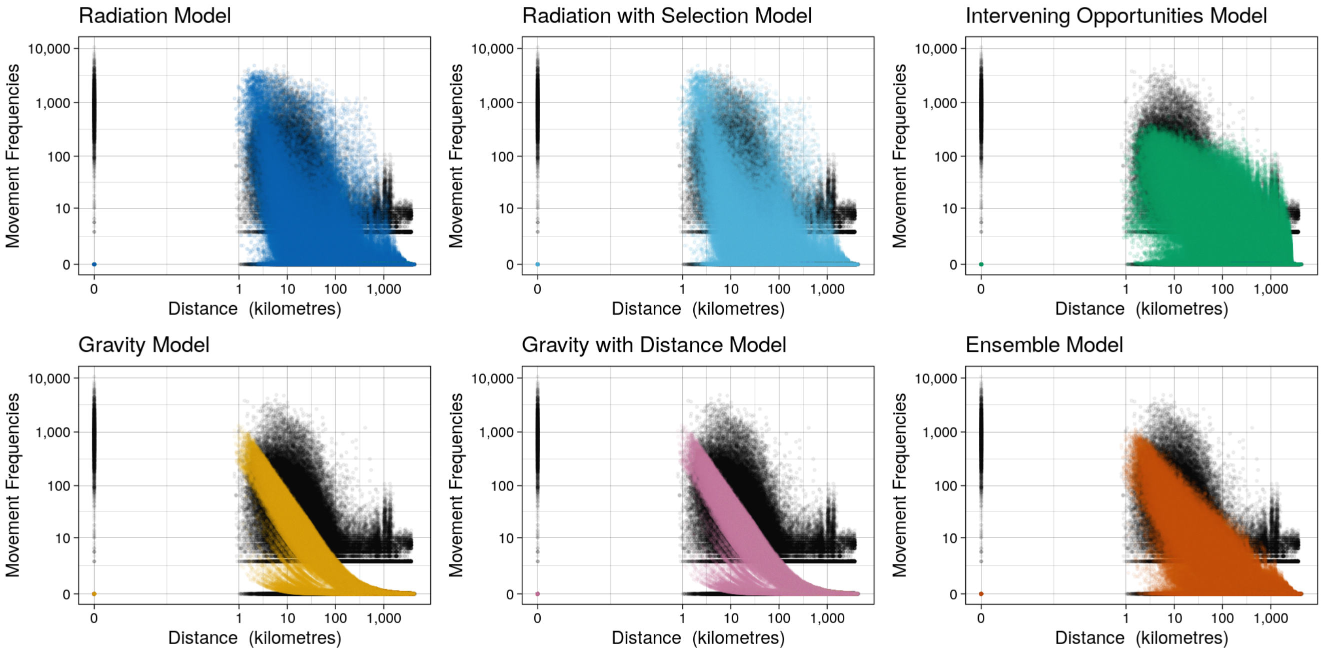

Figure S1. Comparison of predicted frequency of movements by distance (kilometres) between pairs of locations for each model type, based on census data for the whole of Australia. Black points show the raw movement frequencies that were used to train each model. Coloured points show the resulting model predictions. Movement frequency and distance are shown on a log-scale to better highlight differences between model predictions.

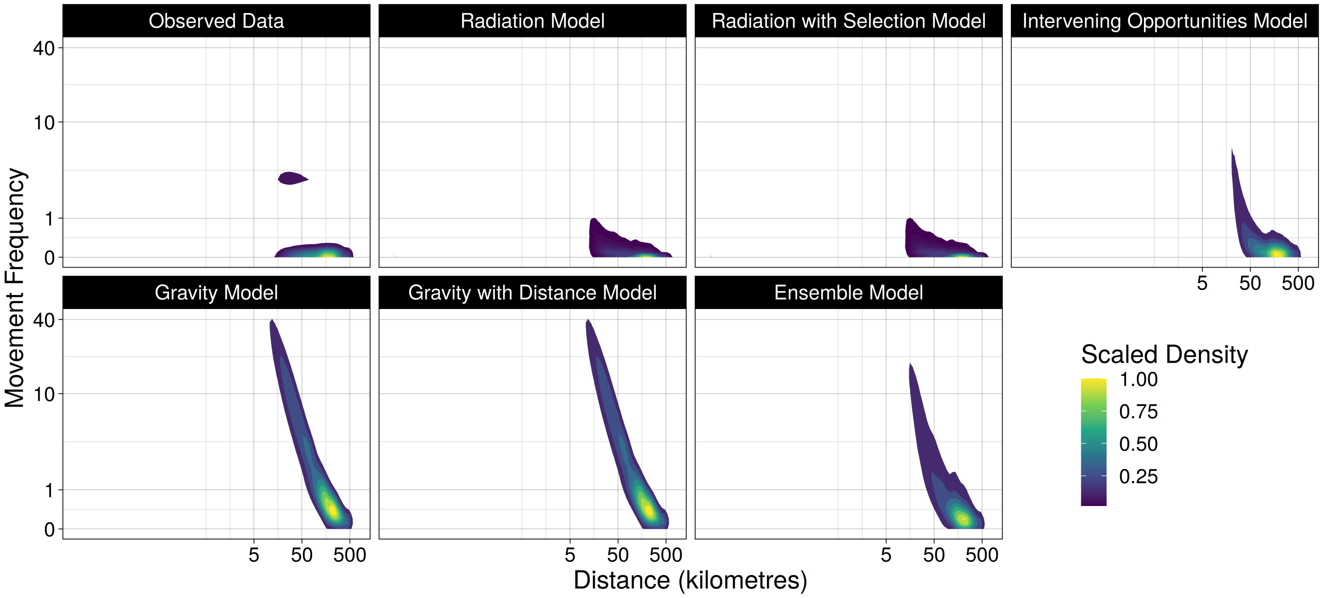

Figure S2. Kernel density estimates of the observed data and predictions from each movement model. Density estimates have been re-scaled to have a maximum of 1. Movement frequency and distance are shown on a log-scale. These provide insight into where the majority of predicted values lie, in comparison to the observed data and provides an alternative view to the same data illustrated in Figure 1 of the manuscript.

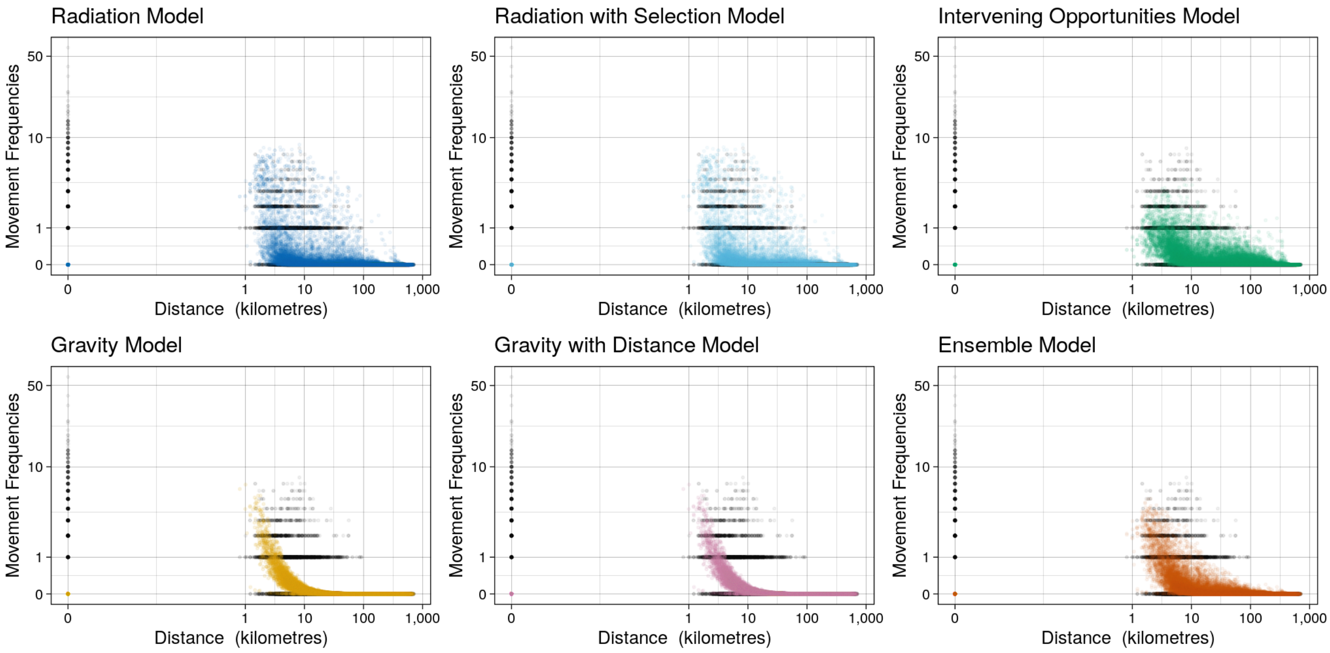

Figure S3. Comparison of predicted frequency of movements by distance (kilometres) between pairs of locations for each model type, based on GPS data for the Australian state of Victoria. Black points show the raw movement frequencies that were used to train each model. Coloured points show the resulting model predictions. Movement frequency and distance are shown on a log-scale to better highlight differences between model predictions.

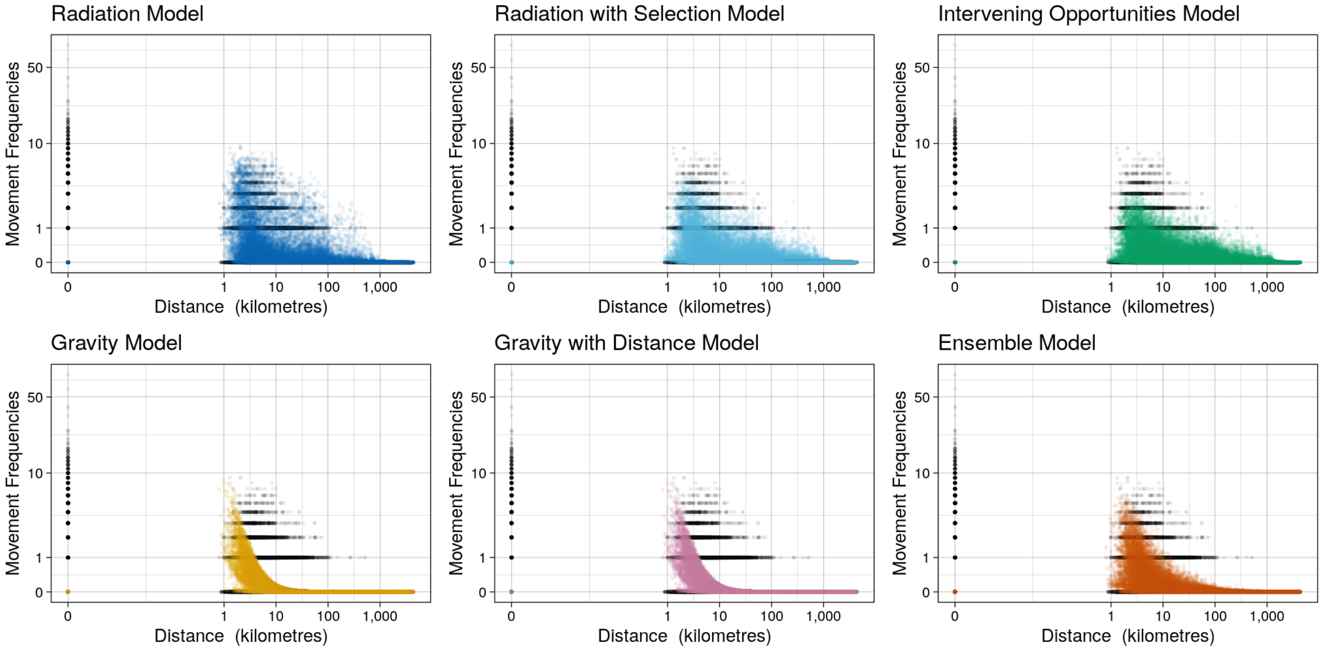

Figure S4. Comparison of predicted frequency of movements by distance (kilometres) between pairs of locations for each model type, based on GPS data for the whole of Australia. Black points show the raw movement frequencies that were used to train each model. Coloured points show the resulting model predictions. Movement frequency and distance are shown on a log-scale to better highlight differences between model predictions.

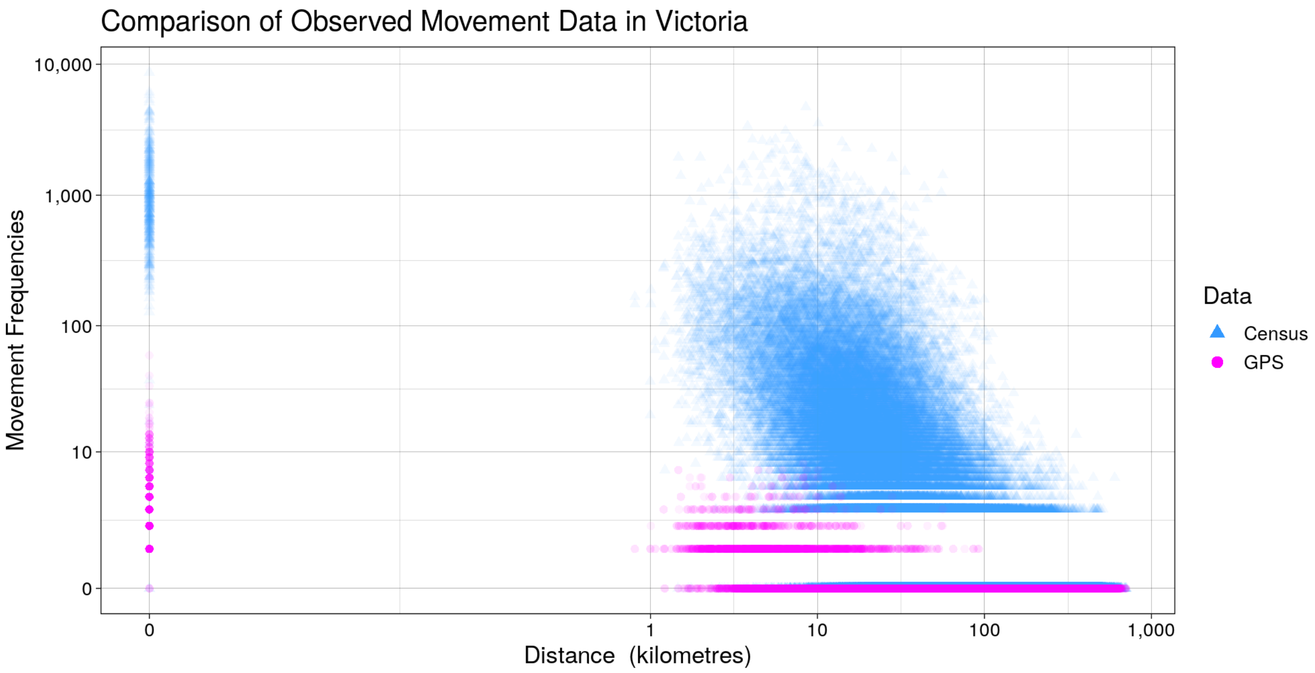

Figure S5. Comparison of observed frequency of movements by distance (kilometres) between pairs of locations for ABS census and GPS data for the Australian state of Victoria. Movement frequency and distance are shown on a log-scale.

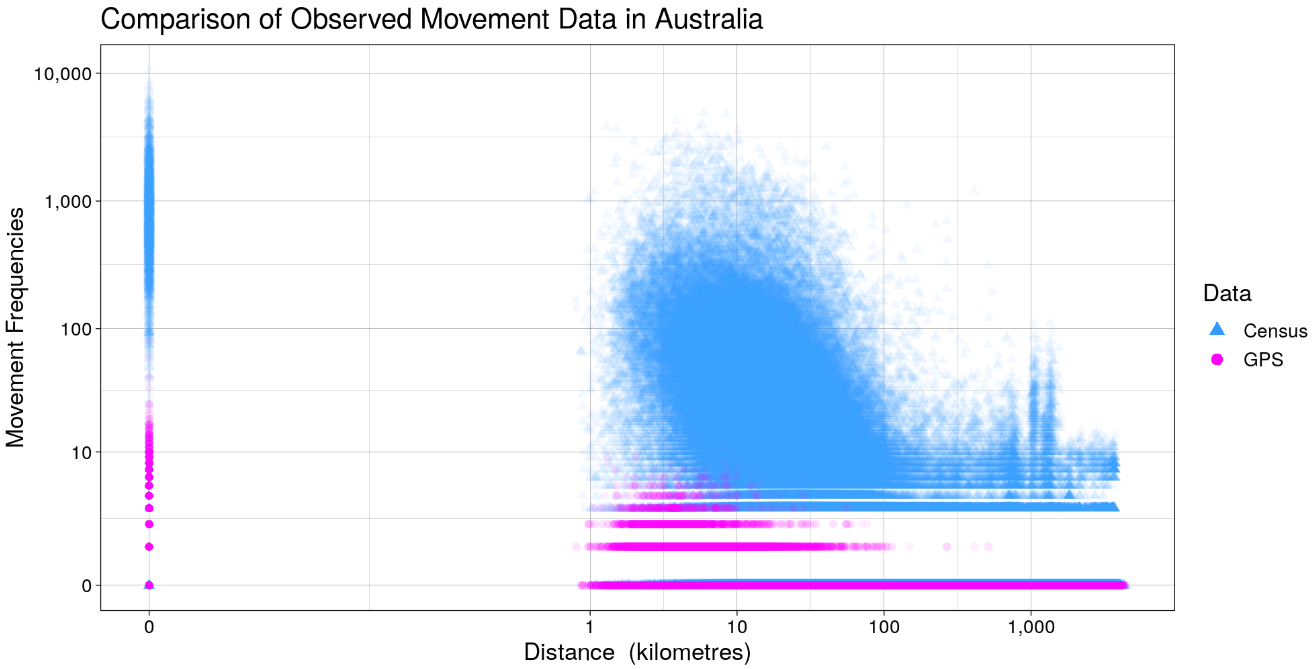

Figure S6. Comparison of observed frequency of movements by distance (kilometres) between pairs of locations for ABS census and GPS data for the whole of Australia. Movement frequency and distance are shown on a log-scale.

Cross validation results

In the following tables, 'Fold *i*' refers to the section of data that was left out of the model training process and the model predictions were generated for 'Fold *i*' in order to see how well the model performs. The Poisson Deviance for ABS census Victorian and Australian scale predictions are given in Tables S1 and S2 respectively. The Poisson Deviance for GPS Victorian and Australian scale predictions are given in Tables S3 and S4 respectively. For each model type, the Poisson deviance was calculated by comparing the predicted movements for entries in each fold which was used to 'test' the model with the corresponding observed values. Tables S5 to S8 provide estimates of model parameters resulting from 5-fold cross validation.

Table S1. Poisson Deviance for 5-fold cross validation using ABS Journey to Work Data: Victorian predictions

Model type	Poisson Deviance						
	Fold 1	Fold 2	Fold 3	Fold 4	Fold 5	Mean	SD
Gravity	870325	860764	891147	831357	872689	865256	19599
Gravity with Distance	870089	860766	891147	806221	872690	860183	28725
Radiation	1630733	1647624	1607337	1521502	1582722	1597984	44060
Radiation with Selection	1630692	1647591	1607299	1521463	1582692	1597947	44061
Intervening Opportunities	824134	848359	855658	795802	826219	830034	21041
Ensemble	692228	696618	713112	608097	700741	682159	37680

Table S2. Poisson Deviance for 5-fold cross validation using ABS Journey to Work Data: Australian predictions

Model type	Poisson Deviance						
	Fold 1	Fold 2	Fold 3	Fold 4	Fold 5	Mean	SD
Gravity	3776069	3590674	3870622	3658351	3860812	3751306	110711
Gravity with Distance	3774959	3590669	3870630	3658349	3860805	3751082	110664
Radiation	6855272	6525158	6782895	6612122	6704687	6696027	117657
Radiation with Selection	6855101	6524986	6782718	6611954	6704509	6695853	117656
Intervening Opportunities	4630580	4267374	4633080	4391866	4565793	4497739	144826
Ensemble	3246282	3090159	3318033	3082473	3297156	3206821	101151

Table S3. Poisson Deviance for 5-fold cross validation using GPS Data: Victorian predictions

Model type	Poisson Deviance						
	Fold 1	Fold 2	Fold 3	Fold 4	Fold 5	Mean	SD
Gravity	3692	3623	3911	3741	3446	3683	152
Gravity with Distance	3692	3623	3911	3741	3446	3683	152
Radiation	3477	3459	3443	3303	3126	3362	133
Radiation with Selection	3477	3459	3443	3303	3126	3361	133
Intervening Opportunities	3078	3182	3191	3048	2843	3068	126
Ensemble	2800	2826	2846	2764	2592	2766	91

Table S4. Poisson Deviance for 5-fold cross validation using GPS Data: Australian predictions

Model type	Poisson Deviance						
	Fold 1	Fold 2	Fold 3	Fold 4	Fold 5	Mean	SD
Gravity	13366	12925	13043	13078	13503	13183	216
Gravity with Distance	13366	12925	13043	13078	13503	13183	216
Radiation	13681	12770	12761	12971	13664	13169	418
Radiation with Selection	11824	11258	11223	11450	11937	11538	292
Intervening Opportunities	12520	11791	11864	11975	12602	12150	341
Ensemble	10890	10373	10362	10561	11002	10638	264

Table S5. Estimated model parameters for 5-fold cross validation using Victorian Census Data

Parameter	Gravity Model						
	Fold 1	Fold 2	Fold 3	Fold 4	Fold 5	Mean	SD
θ	542.712480	499.144061	488.135616	393.037832	524.705711	489.547140	58.031927
α	0.808379	0.785586	0.818792	0.811822	0.789987	0.802913	0.014393
β	0.216192	0.240847	0.212439	0.242781	0.232759	0.229004	0.013989
γ	1.346332	1.339467	1.342627	1.341927	1.341119	1.342295	0.002546

Parameter	Gravity with Distance Model						
	Fold 1	Fold 2	Fold 3	Fold 4	Fold 5	Mean	SD
θ_1	0.000007	0.000007	0.000007	0.000016	0.000007	0.000009	0.000004
α_1	0.802870	0.785559	0.818791	0.831886	0.789861	0.805793	0.019504
β_1	0.228933	0.240874	0.212451	0.200810	0.232725	0.223158	0.016223
γ_1	1.342161	1.339331	1.342489	1.162651	1.341105	1.305547	0.079891
δ	1.000000	0.989783	0.930096	0.058061	0.911853	0.777958	0.404196
θ_2	0.161209	0.108526	0.000001	0.000274	0.000000	0.054002	0.076134
α_2	-14.506976	-16.796865	7.645935	0.086391	1.516347	-4.411033	10.677875
β_2	3.985797	15.505577	-7.779967	0.131058	0.474889	2.463471	8.466650
γ_2	4.845840	7.319459	-17.767873	2.775484	54.404259	10.315434	26.590599

Parameter	Radiation Model						
	Fold 1	Fold 2	Fold 3	Fold 4	Fold 5	Mean	SD
θ	0.315004083	0.3076516	0.313452558	0.314459162	0.304971306	0.311107742	0.004514212

Parameter	Radiation with Selection Model						
	Fold 1	Fold 2	Fold 3	Fold 4	Fold 5	Mean	SD
θ	0.314978	0.307629	0.313428	0.314426	0.304948	0.311082	0.004511
λ	0.200000	0.200000	0.200000	0.200000	0.200000	0.200000	0.000000

Parameter	Intervening Opportunities Model						
	Fold 1	Fold 2	Fold 3	Fold 4	Fold 5	Mean	SD
θ	0.309864	0.310164	0.309060	0.312617	0.309123	0.310166	0.001450
λ	0.000002	0.000002	0.000002	0.000002	0.000002	0.000002	0.000000

Parameter	Ensemble Model						
	Fold 1	Fold 2	Fold 3	Fold 4	Fold 5	Mean	SD
β_0	0.923029	3.169985	0.817638	1.297922	0.850761	1.411867	1.001358
β_1	29.073101	7266.023944	0.269440	-1.158809	-0.404345	1458.760666	3246.384041
β_2	-28.842227	-7266.443796	0.053296	1.371873	0.724193	-1458.627332	3246.693366
β_3	39.724399	4347.731061	721.938934	713.768329	821.819530	1328.996451	1716.044432
β_4	-39.542180	-4347.616698	-721.748239	-713.491169	-821.624113	-1328.804480	1716.084509
β_5	0.432037	0.523710	0.375344	0.295491	0.366879	0.398692	0.085072

Table S6. Estimated model parameters for 5-fold cross validation using Australian Census Data

Parameter	Gravity Model					
	Fold 1	Fold 2	Fold 3	Fold 4	Fold 5	Mean	SD
θ	12102.063283	11166.914934	11714.262980	10831.379919	11769.495746	11516.823372	509.313293
α	0.712173	0.732644	0.718555	0.734691	0.729526	0.725518	0.009716
β	0.041046	0.034399	0.036477	0.033969	0.031932	0.035565	0.003464
γ	1.392653	1.397228	1.391889	1.396035	1.397978	1.395157	0.002737

Parameter	Gravity with Distance Model						
	Fold 1	Fold 2	Fold 3	Fold 4	Fold 5	Mean	SD
θ_1	0.000006	0.000006	0.000007	0.000006	0.000006	0.000006	0.000000
α_1	0.725542	0.732618	0.718554	0.734600	0.729507	0.728164	0.006370
β_1	0.035521	0.034402	0.036486	0.033892	0.031932	0.034447	0.001728
γ_1	1.395046	1.397114	1.391755	1.395938	1.397877	1.395546	0.002380
δ	0.926831	1.000000	0.999945	1.000000	0.999999	0.985355	0.032716
θ_2	0.000016	0.000000	0.000000	0.000000	0.000000	0.000003	0.000007
α_2	-8.595269	2.822226	40.533528	22.677423	17.374756	14.962533	18.851865
β_2	-4.489471	23.165282	-0.860610	1.869413	22.456325	8.428188	13.324186
γ_2	341.903571	-126.802012	-148.718315	-352.821063	-158.574976	-89.002559	257.446785

Parameter	Radiation Model						
	Fold 1	Fold 2	Fold 3	Fold 4	Fold 5	Mean	SD
θ	0.306963	0.310690	0.306078	0.307311	0.307139	0.307636	0.001772

Parameter	Radiation with Selection Model						
	Fold 1	Fold 2	Fold 3	Fold 4	Fold 5	Mean	SD
θ	0.306958	0.310683	0.306072	0.307305	0.307134	0.307630	0.001771
λ	0.204873	0.201590	0.200013	0.200144	0.200683	0.201461	0.002006

Parameter	Intervening Opportunities Model						
	Fold 1	Fold 2	Fold 3	Fold 4	Fold 5	Mean	SD
θ	0.306711	0.310133	0.305162	0.308583	0.306580	0.307434	0.001938
λ	0.000002	0.000002	0.000002	0.000002	0.000002	0.000002	0.000000

Parameter	Ensemble Model						
	Fold 1	Fold 2	Fold 3	Fold 4	Fold 5	Mean	SD
β_0	1.147783	2.058024	1.652175	-0.566816	1.570570	1.172347	1.024516
β_1	-27.549991	4000.096214	2254.309500	-4643.331072	2766.619779	870.028886	3410.036553
β_2	27.929339	-3999.948091	-2254.046321	4644.106838	-2766.342125	-869.660072	3410.275776
β_3	-16.132971	53.502781	52.937247	65.056935	50.684692	41.209737	32.539604
β_4	16.348850	-53.299448	-52.740149	-64.869276	-50.484834	-41.008971	32.548818
β_5	0.204413	0.205276	0.213030	0.302958	0.201745	0.225484	0.043512

Table S7. Estimated model parameters for 5-fold cross validation using Victorian GPS Data

Parameter	Gravity Model						
	Fold 1	Fold 2	Fold 3	Fold 4	Fold 5	Mean	SD
θ	25.384241	21.837919	20.532605	7.268214	34.260003	21.856597	9.757856
α	0.755681	0.732994	0.747706	0.755619	0.688272	0.736054	0.028268
β	0.668351	0.699855	0.704591	0.808617	0.700702	0.716423	0.053555
γ	2.114622	2.107197	2.123654	2.125910	2.109196	2.116116	0.008403

Parameter	Gravity with Distance Model						
	Fold 1	Fold 2	Fold 3	Fold 4	Fold 5	Mean	SD
θ_1	0.000000	0.000000	0.000000	0.000000	0.000000	0.000000	0.000000
α_1	0.756136	0.732701	0.747675	0.755591	0.688233	0.736067	0.028364
β_1	0.669128	0.699632	0.704561	0.808537	0.700645	0.716501	0.053370
γ_1	2.114680	2.107104	2.123498	2.125734	2.109042	2.116012	0.008370
δ	1.000000	1.000000	1.000000	1.000000	1.000000	1.000000	0.000000
θ_2	0.000016	0.000000	0.000084	0.000024	0.051650	0.010355	0.023085
α_2	2.217233	43.489575	-17.954174	0.107413	-0.822735	5.407462	22.765636
β_2	-1.909653	-40.505792	18.701159	-0.059390	0.004635	-4.753808	21.685879
γ_2	65.833893	40.862476	42.676555	26.653905	70.097111	49.224788	18.260398

Parameter	Radiation Model						
	Fold 1	Fold 2	Fold 3	Fold 4	Fold 5	Mean	SD
θ	0.000686	0.000679	0.000678	0.000686	0.000680	0.000682	0.000004

Parameter	Radiation with Selection Model						
	Fold 1	Fold 2	Fold 3	Fold 4	Fold 5	Mean	SD
θ	0.000686	0.000679	0.000678	0.000686	0.000680	0.000682	0.000004
λ	0.200000	0.200000	0.200000	0.200000	0.200000	0.200000	0.000000

Parameter	Intervening Opportunities Model						
	Fold 1	Fold 2	Fold 3	Fold 4	Fold 5	Mean	SD
θ	0.000675	0.000682	0.000672	0.000681	0.000688	0.000680	0.000006
λ	0.000008	0.000008	0.000008	0.000008	0.000008	0.000008	0.000000

Parameter	Ensemble Model						
	Fold 1	Fold 2	Fold 3	Fold 4	Fold 5	Mean	SD
β_0	0.323295	0.319331	-0.126461	-0.086980	-0.549702	-0.024103	0.363771
β_1	146.069958	-370.370776	3988.255814	3856.287511	10321.942639	3588.437029	4274.744058
β_2	-145.821481	370.627518	-3988.298730	-3856.359020	-10322.450208	-3588.460384	4275.055525
β_3	-95.175511	-92.090215	-91.550018	-91.245226	-94.106685	-92.833531	1.719624
β_4	95.488359	92.393200	91.845396	91.553120	94.426560	93.141327	1.726406
β_5	0.372478	0.389898	0.389353	0.376865	0.354898	0.376698	0.014383

Table S8. Estimated model parameters for 5-fold cross validation using Australian GPS Data

Parameter	Gravity Model						
	Fold 1	Fold 2	Fold 3	Fold 4	Fold 5	Mean	SD
θ	366.546374	386.255897	377.210694	320.708387	358.980130	361.940296	25.270943
α	0.567363	0.558751	0.525975	0.546893	0.540320	0.547860	0.016091
β	0.527396	0.528001	0.563763	0.560560	0.558337	0.547611	0.018281
γ	2.059716	2.056153	2.057425	2.057797	2.061151	2.058449	0.001978

Parameter	Gravity with Distance Model						
	Fold 1	Fold 2	Fold 3	Fold 4	Fold 5	Mean	SD
θ_1	0.000000	0.000000	0.000000	0.000000	0.000000	0.000000	0.000000
α_1	0.567434	0.558594	0.525912	0.546551	0.540301	0.547759	0.016116
β_1	0.528064	0.527896	0.563830	0.560161	0.558346	0.547659	0.018073
γ_1	2.059437	2.056096	2.057072	2.057776	2.060957	2.058268	0.001936
δ	0.736052	0.923615	0.989883	0.877521	0.968909	0.899196	0.100953
θ_2	0.000000	0.000000	0.000000	0.000000	0.000000	0.000000	0.000000
α_2	10.246065	6.819334	8.022712	4.402432	-0.247364	5.848636	4.008085
β_2	6.276850	0.417435	0.615795	-1.440286	-0.582966	1.057366	3.032180
γ_2	511.935000	54.493099	57.045847	45.301051	42.771883	142.309376	206.714173

Parameter	Radiation Model						
	Fold 1	Fold 2	Fold 3	Fold 4	Fold 5	Mean	SD
θ	0.000591	0.000598	0.000597	0.000592	0.000590	0.000593	0.000004

Parameter	Radiation with Selection Model						
	Fold 1	Fold 2	Fold 3	Fold 4	Fold 5	Mean	SD
θ	0.000591	0.000598	0.000595	0.000595	0.000589	0.000594	0.000004
λ	0.999960	0.999960	0.999960	0.999960	0.999960	0.999960	0.000000

Parameter	Intervening Opportunities Model						
	Fold 1	Fold 2	Fold 3	Fold 4	Fold 5	Mean	SD
θ	0.000590	0.000597	0.000594	0.000595	0.000589	0.000593	0.000003
λ	0.000009	0.000009	0.000009	0.000009	0.000009	0.000009	0.000000

Parameter	Ensemble Model						
	Fold 1	Fold 2	Fold 3	Fold 4	Fold 5	Mean	SD
β_0	0.319089	0.450567	0.082933	0.315517	0.317712	0.297164	0.132917
β_1	0.499518	-1226.486339	1279.527353	-3.822397	3.851779	10.713983	886.131301
β_2	-0.184051	1226.851794	-1279.414453	4.142220	-3.535845	-10.428067	886.221281
β_3	-0.060965	-0.141012	-0.173493	-0.107246	-0.087306	-0.114004	0.044272
β_4	0.634474	0.790819	0.826235	0.705570	0.677866	0.726992	0.079616
β_5	0.151634	0.121927	0.120455	0.135735	0.142286	0.134408	0.013333
